# *R*-Flurbiprofen Traps Prostaglandins within Cells by Inhibition of Multidrug Resistance-Associated Protein-4

**DOI:** 10.3390/ijms18010068

**Published:** 2016-12-30

**Authors:** Ivonne Wobst, Lisa Ebert, Kerstin Birod, Marthe-Susanna Wegner, Marika Hoffmann, Dominique Thomas, Carlo Angioni, Michael J. Parnham, Dieter Steinhilber, Irmgard Tegeder, Gerd Geisslinger, Sabine Grösch

**Affiliations:** 1 Pharmazentrum frankfurt, ZAFES, Institute for Clinical Pharmacology, Goethe-University Frankfurt, Theodor-Stern-Kai 7, 60590 Frankfurt/Main, Germany; mail@iwobst.de (I.W.); k.birod@med.uni-frankfurt.de (K.B.); marthewegner@me.com (M.-S.W.); thomas@med.uni-frankfurt.de (D.T.); Angioni@em.uni-frankfurt.de (C.A.); tegeder@em.uni-frankfurt.de (I.T.); geisslinger@em.uni-frankfurt.de (G.G.); 2 Fraunhofer Institute for Molecular Biology and Applied Ecology IME, Project Group Translational Medicine and Pharmacology TMP, Theodor-Stern-Kai 7, 60590 Frankfurt am Main, Germany; ebert.lisa@web.de (L.E.); Michael.Parnham@ime.fraunhofer.de (M.J.P.); 3 Institute of Pharmaceutical Chemistry, ZAFES, Johann Wolfgang Goethe-University Frankfurt, Max-von-Laue-Str. 9, D-60438 Frankfurt, Germany; steinhilber@em.uni-frankfurt.de

**Keywords:** flurbiprofen, MRP4, cPLA_2_, PGE_2_

## Abstract

*R*-flurbiprofen is the non-COX-inhibiting enantiomer of flurbiprofen and is not converted to *S*-flurbiprofen in human cells. Nevertheless, it reduces extracellular prostaglandin E_2_ (PGE_2_) in cancer or immune cell cultures and human extracellular fluid. Here, we show that *R*-flurbiprofen acts through a dual mechanism: (i) it inhibits the translocation of cPLA_2α_ to the plasma membrane and thereby curtails the availability of arachidonic acid and (ii) *R*-flurbiprofen traps PGE_2_ inside of the cells by inhibiting multidrug resistance–associated protein 4 (MRP4, ABCC4), which acts as an outward transporter for prostaglandins. Consequently, the effects of *R*-flurbiprofen were mimicked by RNAi-mediated knockdown of MRP4. Our data show a novel mechanism by which *R*-flurbiprofen reduces extracellular PGs at physiological concentrations, particularly in cancers with high levels of MRP4, but the mechanism may also contribute to its anti-inflammatory and immune-modulating properties and suggests that it reduces PGs in a site- and context-dependent manner.

## 1. Introduction

Prostaglandins (PGs) are key mediators in the regulation of pain and inflammation, mucosal health, tumor growth, angiogenesis, blood pressure, kidney function, ocular pressure, neurovascular coupling, and glial and neuronal functions [1]. They are produced in a multi-step process involving cytosolic phospholipase A_2_ (cPLA_2_), cyclooxygenase-1 or -2 (COX-1/-2) and subsequent prostaglandin synthases. PGs poorly pass membranes and therefore use cell membrane–bound transporters for cellular release. An important membrane transporter for the export of PGs from cells to the extracellular space is the multidrug resistance protein 4 (MRP4), also known as ATP-binding cassette transporter (ABCC4). Once released, PGs bind to and activate specific PG receptors in an autocrine or paracrine manner. 

PGs play a distinct role in many pathophysiological processes such as pain, inflammation, cancer and autoimmunity. Pharmacologically, PG release is commonly inhibited by blocking COX-1 and/or COX-2 with non-steroidal anti-inflammatory drugs (NSAIDs), such as flurbiprofen. Flurbiprofen has been used to treat patients with rheumatoid arthritis for more than 40 years [2]. It belongs to the class of 2-arylpropionic acids and is the 1:1 racemate of the *S*- and *R*-enantiomers. *S*-flurbiprofen inhibits COX-1 and -2, but *R*-flurbiprofen is inactive as a COX-inhibitor. Nevertheless, both enantiomers have anti-nociceptive, anti-inflammatory and anti-cancer effects [3,4,5,6]. *R*-flurbiprofen is not inverted to *S*-flurbiprofen in humans and minimally in rats [5,7]. Hence, effects observed in humans or rats or human cells cannot be attributed to COX inhibition.

About 30 years ago flurbiprofen was found to reduce the risk of colon cancer, which was ascribed mainly to its inhibitory effect on COX [8,9]. In further studies, we demonstrated that the anti-proliferative effects of both *R*- and *S*-flurbiprofen in colon cancer cells were mediated at least in part via COX-independent mechanisms [3,10]. *R*- and *S*-flurbiprofen regulate signaling pathways converging on c-Jun N-terminal kinase (JNK) [3], nuclear factor kappa B (NF-κB) [4] or p53 [10]. Other NSAIDS also reduce the risk of colon cancer, but are not used as “anti-cancer drugs” because they cause gastrointestinal and cardiovascular side effects, mainly caused by inhibition of COX-1- and COX-2-mediated generation of prostacyclin, and PGE_2_, respectively [11,12,13]. Hence, *R*-flurbiprofen has some advantages. *R*-flurbiprofen inhibited the organ release of prostaglandins ex vivo, e.g., from the lung and brain of the rat, but not in the mucosa of the small intestine at concentrations of 25 mg/kg [14,15]. *R*-flurbiprofen also reduced PG levels in blister fluid [16] and the microdialysates of the rat brain or spinal cord [14]. Hence, it obviously reduces extracellular prostaglandin levels without affecting COXs. In the present study, we assessed the underlying molecular mechanisms using an in vitro model of human cancer cell lines, with differential expression of MRP4, based on the hypothesis that *R*-flurbiprofen, which has been shown to interfere with organic anion transporters (OATs), may target MRP4-mediated PG transport.

## 2. Results

### 2.1. R-Flurbiprofen Reduces Prostaglandin Levels

**Figure 1:**
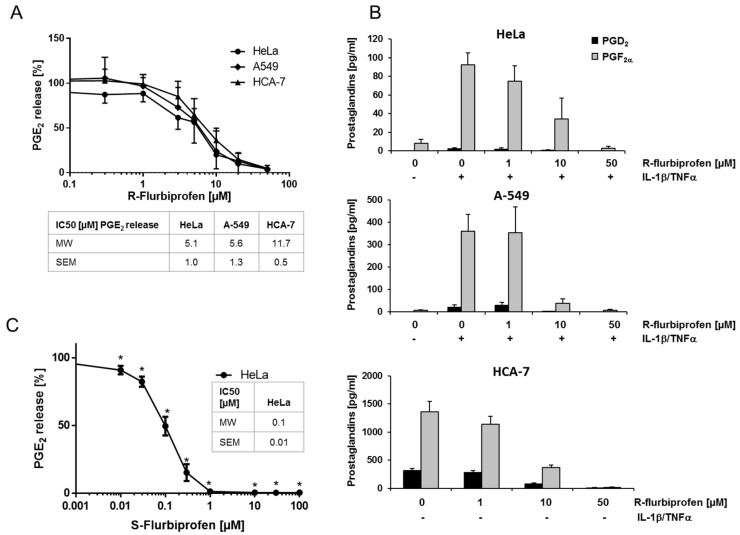
PGE_2_ in cell supernatant of human cancer cell lines: Cells were treated either with 0.1% DMSO (control), *S*-flurbiprofen (0.01–10 µM) or *R*-flurbiprofen (0.03–50 µM) and, simultaneously, with IL-1β (2 ng/mL) for stimulation of A-549 cells or IL-1β (1 ng/mL) + TNFα (5 ng/mL) for stimulation of HeLa cells. HCA-7 cells were pre-incubated with medium containing either DMSO (0.1%) or flurbiprofen for 1 h and this medium was replaced with fresh medium and the same additions. (**A**) *R*-flurbiprofen inhibited PGE_2_ production by all three cell lines at concentrations between 1 and 50 µM. Means ± S.E.M. of four independent experiments; (**B**) *R*-flurbiprofen also inhibited the release of PGD_2_ and PGF_2α_ from HeLa, A-549 and HCA-7 cells in a concentration-dependent manner. Means ± S.E.M. of three independent experiments; (**C**) *S*-flurbiprofen is 50-fold more potent than *R*-flurbiprofen in inhibiting PGE_2_ production in HeLa cells with an IC_50_ of about 100 nM. Means ± S.E.M. of three independent experiments. Significant *p* values are shown as: * *p* ≤ 0.05.

Several studies have shown that *R*-flurbiprofen inhibits PG levels in different tissues, despite its lack of COX-inhibiting activity. We confirmed these effects in cancer cell supernatants of human cervical (HeLa), lung (A-549) and colon (HCA-7) cancer cell lines. HeLa and A-549 cells produce only marginal basal amounts of PGE_2_ (17.4 ± 2.2 pg/mL (HeLa) and 231 ± 73 pg/mL (A549)). Therefore, these cells were stimulated with IL-1β (A-549) or IL-1β + TNFα (HeLa) for 16 h, resulting in a strong increase of PGE_2_ in these cells (5807 ± 110 pg/mL (A-549), 4617 ± 537 pg/mL (HeLa)). HCA-7 cells produce high amounts of PGE_2_ without stimulation (6812 ± 1284 pg/mL). *R*-flurbiprofen reduced PGE_2_ concentrations in supernatants of all three cell lines with IC_50_ values of 5.1 µM (HeLa), 5.6 µM (A-549) and 11.7 µM (HCA-7), respectively (Figure 1A). PGD_2_ and PGF_2α_ were also reduced in the supernatants of all three cell lines after *R*-flurbiprofen treatment (Figure 1B), suggesting that *R*-flurbiprofen unspecifically affects all PGs. As expected, *S*-flurbiprofen, which is a potent COX-1/2 inhibitor, was more potent with an IC_50_ of 0.1 µM (Figure 1C). The effects of *R*-flurbiprofen are still clinically relevant because patients received up to 800 mg (twice a day) [17], leading to plasma concentrations of about 185 µM [18].

### 2.2. Inhibition of PG Release Is Not Mediated through COX-1/2- or mPGES-1

**Figure 2:**
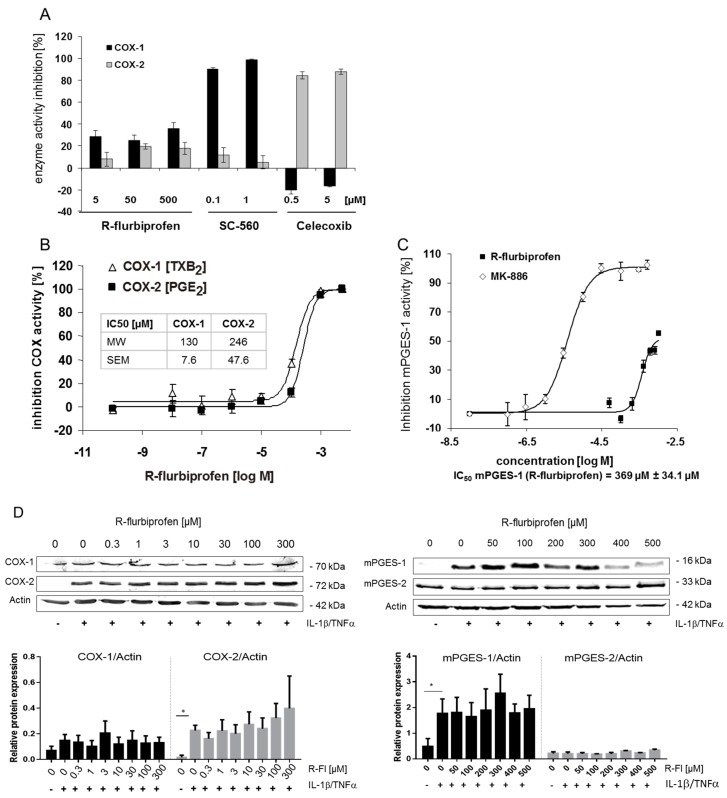
(**A**) COX inhibitor screening assay: in vitro inhibition of purified COX-1 and COX-2 protein by *R*-flurbiprofen, SC-560, and celecoxib. *R*-flurbiprofen inhibited COX-1 and COX-2 activity only marginally, without concentration dependency. Means ± S.E.M. of four independent experiments; (**B**) Human whole-blood assay in vitro: human blood was pre-incubated with *R*-flurbiprofen or vehicle (DMSO) and subsequently stimulated either with Ca^2+^-ionophore (20 μM A23187) for induction of COX-1 activity, resulting in the formation of thromboxane B_2_ (TXB_2_), or with LPS (5 mg/mL) for induction of COX-2 activity, resulting in the formation of PGE_2_. IC_50_ values were calculated using a sigmoid Emax model. Data presented are means ± S.E.M. of four independent experiments; (**C**) mPGES-1 activity assay: mPGES-1 activity was measured in the microsomal fraction of HeLa cells in vitro. *R*-flurbiprofen only marginally affected mPGES-1 activity, with an IC_50_ of about 370 µM. In contrast, MK-886 inhibited mPGES-1 activity with an IC_50_ of 4 µM. IC_50_ values were calculated using a sigmoid Emax model. Data presented are means ± S.E.M. of four independent experiments; (**D**) Western blot analysis of COX-1 and COX-2 (whole cell lysate) and mPGES-1/2 (microsomal fraction). One representative experiment out of three is shown, as well as the quantification of three Western blots. Data presented are means ± S.E.M. of three independent experiments; statistical analysis was done with one-way ANOVA. Significant *p* values are shown as: * *p* ≤ 0.05.

We assessed the effect of *R*-flurbiprofen on the activity of purified COX-1, COX-2 and mPGES enzymes on COX-1 and COX-2 in the whole-blood assay and on protein expression in HeLa cells. *R*-flurbiprofen achieved a maximum inhibition of purified COX-1 and COX-2 of 30% at 500 µM (Figure 2A). The positive controls SC-560 (COX-1) [19] and celecoxib (COX-2) were much more potent. In contrast, in the whole-blood assay, *R*-flurbiprofen inhibited the release of thromboxane B_2_ (readout for COX-1 inhibition) as well as PGE_2_ (readout for COX-2 inhibition) with an IC_50_ (COX inhibition) of 130 and 246 µM, respectively (Figure 2B). The activity of purified mPGES-1 was inhibited by *R*-flurbiprofen with an IC_50_ of 369 µM (Figure 2C), i.e., at much higher concentrations than PG inhibition in cell culture supernatants. Treatment with *R*-flurbiprofen had no effect on COX-1, COX-2, mPGES-1 or mPGES-2 protein expression, up to a concentration of 300 µM (Figure 2D). *R*-flurbiprofen did not affect cPGES protein levels (data not shown).

### 2.3. R-Flurbiprofen Targets Alternative Candidates in the PG Pathway

**Figure 3:**
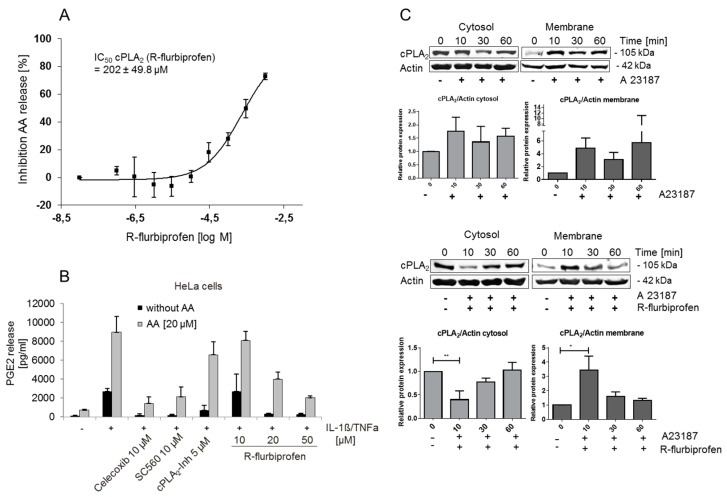
(**A**) cPLA_2α_ activity assay: for the in vitro cPLA_2α_ activity assay, small unilamellar vesicles containing 1-palmitoyl-2 arachidonyl-sn-glycero-3-phosphocholine (PAPC) and 1-palmitoyl-2-oleoyl-sn-glycerol (POG) 2:1 were prepared and incubated with cPLA_2α_ enzyme isolated from Sf9-insekt cells. The IC_50_ value was calculated using a sigmoid Emax model. Data presented are means ± S.E.M. of four independent experiments; (**B**) PGE_2_ in cell supernatant of HeLa cells with or without arachidonic acid addition: HeLa cells were stimulated with IL-1β (1 ng/mL) and TNFα (5 ng/mL) and simultaneously treated with *R*-flurbiprofen (10, 20, 50 µM), 10 µM celecoxib, 10 µM SC-560 or 5 µM cPLA_2α_ inhibitor. Additionally, 20 µM arachidonic acid (AA) was added to the cells, and after 16 h incubation, the PGE_2_ level in supernatants was determined. Means ± S.E.M. of three independent experiments; (**C**) Western blot analysis of cPLA_2α_ in A549 cytosolic and membrane fractions: A549 cells were stimulated with the Ca^2+^-ionophore (A23187) for 10, 30 or 60 min and additionally treated with 100 µM *R*-flurbiprofen. Actin was used as a loading control. One representative experiment out of three is shown, as well as the quantification of the three Western blots. Data presented are means ± S.E.M. of three independent experiments; statistical analysis was done with one-way ANOVA. Significant *p* values are shown as: ** *p* ≤ 0.01, * *p* ≤ 0.05.

The results presented above did not explain the *R*-flurbiprofen–mediated reduction of PGs in cell culture supernatants. We therefore assessed alternative targets. PG production starts with the release of arachidonic acid from phospholipids in cell membranes. The most important enzyme responsible for the release of arachidonic acid (AA) from phospholipids is the cytoplasmic phospholipase A_2α_ (cPLA_2α_) [20]. *R*-flurbiprofen inhibited the activity of purified recombinant cPLA_2α_ at high concentrations with an IC_50_ of 202 µM (Figure 3A). We also assessed cPLA_2α_ activity in cell culture indirectly by measuring PGE_2_ formation after arachidonic acid (AA) addition (20 µM) to the cells to uncouple PGE_2_ production from endogenous AA release. For this assay, we used stimulated HeLa cells and incubated them with celecoxib, SC-560, cPLA_2_α inhibitor and different concentrations of *R*-flurbiprofen (Figure 3B). AA prevented the inhibition of PGE_2_ release by the cPLA_2α_ inhibitor but the addition of AA failed to prevent the inhibition of PGE_2_ release after celecoxib or SC-560 treatment, as expected. However, AA abolished the effects of 10 µM *R*-flurbiprofen, but could only in part reverse the effect at higher *R*-flurbiprofen concentrations of 20 and 50 µM. Hence, the effects of *R*-flurbiprofen depended in part on the release of AA from phospholipids by cPLA_2α_ in intact cells, but not in cell-free assays, suggesting that *R*-flurbiprofen did not affect the catalytic activity of cPLA_2α_ but rather its targeting to the plasma membrane.

*R*-flurbiprofen did not affect the total cPLA_2α_ protein expression as assessed by Western blot analysis (data not shown), but inversely regulated the expression in cytoplasmic and membrane compartments. cPLA_2α_ translocation from the cytosol to membranes is increased by intracellular concentrations of calcium [Ca^2+^]_i_. Ca^2+^ binds to the N-terminal C2 domain of cPLA_2α_ which is a calcium and phospholipid binding domain that functions primarily to promote interactions with membranes [21]. We used A549 cells for this experiment because baseline membrane targeting of cPLA_2α_ is low in these cells. They were stimulated with calcium-ionophore A23178 with/without *R*-flurbiprofen (Figure 3C). A23178 resulted in a translocation of cPLA_2α_ from the cytosol to cell membranes, which was temporally inhibited by *R*-flurbiprofen. Hence, *R*-flurbiprofen inhibited cPLA_2α_ activity in a cellular context by blocking its Ca^2+^-induced translocation to cell membranes. These effects, however, accounted only for a part of the *R*-flurbiprofen–mediated PG inhibition, as Figure 3B indicates; therefore, other additional mechanisms contribute to PG inhibition. 

### 2.4. R-Flurbiprofen Traps PGs Inside of Cells

PGs are poorly membrane permeable and are transported via specific membrane proteins. The export from cells is mainly mediated by the multidrug resistance-associated protein 4 (MRP4) [22] whereas the reimport is dependent on the prostaglandin transporter (PGT) [23,24].

**Figure 4:**
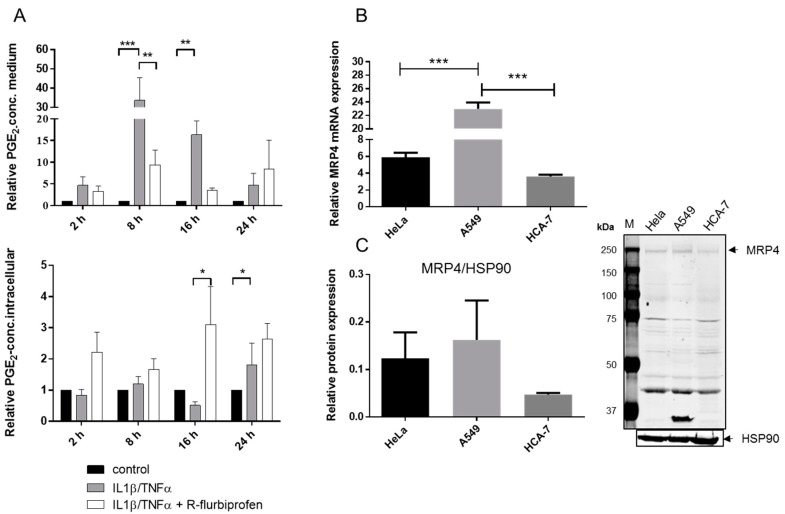
(**A**) Extracellular and intracellular PGE_2_ concentrations of stimulated HeLa cells: HeLa cells were stimulated with IL-1β (1 ng/mL) and TNFα (5 ng/mL) for 16 h and additionally treated with 10 µM *R*-flurbiprofen. PGE_2_ in the supernatant and in full cell lysate was determined by nano-LC-MS/MS. Means ± S.E.M. of four independent experiments; (**B**) mRNA expression of MRP4 in unstimulated cancer cells: mRNA from untreated HeLa, A549, and HCA-7 cells was prepared and MRP4 expression detected by quantitative RT-PCR. Means ± S.E.M. of three independent experiments, statistical analysis was done with one-way ANOVA; (**C**) Western blot analysis of MRP4 in unstimulated cancer cells: protein extracts from untreated HeLa, A549, and HCA-7 cells were prepared, separated by SDS-PAGE and electro-blotted onto a nitrocellulose membrane. MRP4 expression was detected by an anti-MRP4-antibody. HSP90 was used as the loading control. One representative experiment out of three is shown as well as the quantification of the three Western blots. Data presented are means ± S.E.M. of three independent experiments; statistical analysis was done with one-way ANOVA. Significant *p* values are shown as: *** *p* ≤ 0.001, ** *p* ≤ 0.01, * *p* ≤ 0.05.

To investigate whether *R*-flurbiprofen inhibits PG release, we measured intra- and extra-cellular PGE_2_ levels in IL1β-/TNFα-stimulated HeLa cells after *R*-flurbiprofen treatment (Figure 4A). Treatment of HeLa cells with IL1β/TNFα increased extracellular PGE_2_ already after 2 h. Co-treatment with 10 µM *R*-flurbiprofen increased intracellular PGE_2_ levels and this was accompanied by a slight decrease in extracellular PGE_2_. Similar effects were observed at 8–24 h, and were most pronounced at 16 h. Hence, *R*-flurbiprofen blocked the export of PGE_2_, possibly by interfering with the membrane transporter MRP4. The flurbiprofen racemate was previously shown to inhibit MRP4 in the concentration range of 5–50 µM [25], fitting well to the concentration range that inhibited extracellular PGs in our experiments. Figure 4B,C show the expression of MRP4 in our cells; it was expressed highest in A549. 

**Figure 5:**
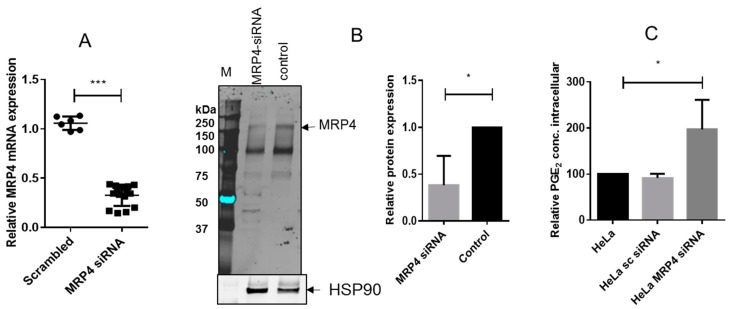
(**A**) MRP4 mRNA expression in HeLa cells after transfection of scrambled or MRP4 siRNA. Means ± S.E.M. of three independent experiments, determined in triplicate, are shown. Statistical analysis was done with *t*-test; (**B**) Western blot analysis of MRP4 in MRP4 siRNA-treated HeLa cells and control cells: HSP90 was used as the loading control. One representative experiment out of three is shown, as well as the quantification of the three Western blots (MRP4/HSP90). Means ± S.E.M. of three independent experiments. Statistical analysis was done with *t*-test; (**C**) Intracellular PGE_2_ concentrations of stimulated HeLa cells after MRP4 siRNA treatment. HeLa cells were transfected with MRP4 siRNA or scrambled siRNA, and 24 h later stimulated with IL-1β (1 ng/mL) and TNFα (5 ng/mL) for 16 h. Intracellular PGE_2_ was determined by nano-LC-MS/MS. Means ± S.E.M. of three experiments. Statistical analysis was done with one-way ANOVA. Significant *p* values are shown as: *** *p* ≤ 0.001, * *p* ≤ 0.05.

To assess if the effects of *R*-flurbiprofen depend on MRP4, we silenced MRP4 via siRNA transfection (Figure 5A mRNA and B protein). The knockdown resulted in intracellular trapping of PGE_2_ as expected (Figure 5C), indicating that PGE_2_ export from our cells indeed depended on MRP4 and suggesting that it was the major *R*-flurbiprofen target. 

## 3. Discussion

We show in the present study that *R*-flurbiprofen traps PGE_2_ inside of cells by inhibition of the multidrug resistance–associated protein, MRP4, which acts as a PG exporter. In addition, *R*-flurbiprofen mildly blocks the membrane translocation of cPLA_2_, which is triggered by intracellular calcium. Thereby, *R*-flurbiprofen limits the availability of the substrate, arachidonic acid, for COXs. 

*R*-flurbiprofen has long been considered as the “inactive” enantiomer of flurbiprofen [26], owing to its failure to inhibit the catalytic activity of COXs. However, *R*-flurbiprofen has anti-nociceptive, anti-inflammatory, immune-modulatory and anti-carcinogenic effects and mildly protects animals in Alzheimer’s models from a decline in cognitive functions [10,14,18,27,28,29,30,31,32,33,34,35].

The anti-carcinogenic effects of *R*-flurbiprofen in mice have been associated in part with the activation of c-Jun-N-terminal kinase, the accumulation of p53 and p75 (NTR) and/or the activation of protein kinase B (PKB/AKT) [3,10,36,37]. The analgesic effect of *R*-flurbiprofen is mediated in part via restoration of endogenous cannabinoids [27], mediated via inhibition of the degradation via FAAH or oxidatively via COXs [38,39].

Our data now show that *R*-flurbiprofen inhibits the PG pathway in part by inhibition of cPLA_2α_ translocation to cell membranes, which occurred at a concentration of 100 µM, in the range of those found in the plasma of patients treated with *R*-flurbiprofen [18]. cPLA_2_ is ubiquitously expressed and responsible for the very first step in prostaglandin synthesis. Therefore, cPLA_2_ inhibition is relevant for most cells.

Additionally, we found that *R*-flurbiprofen inhibits the MRP4-mediated export of PGs, and this effect is tissue- and possibly context-dependent because MRP4 expression is regulated by various stimuli such as estrogen [40] or xenobiotics in an age-dependent manner [41]. 

PGs are known to be substrates of human organic anion transporters such as OAT1, OAT3, the prostaglandin transporter (PGT) and MRP4, which import or export PGs, respectively. Inhibition of OAT1, OAT3 and MRP4 by *R*- and *S*-flurbiprofen was previously reported [25,42]. However, *S*-flurbiprofen is somewhat more effective [25], but, importantly, *R*-flurbiprofen is not toxic whereas *S*-flurbiprofen leads to substantial damage of the gastrointestinal mucosa. MRP4 is highly expressed whereas PGT is mostly downregulated in various cancers, including the cancer cell lines used in the present study [43]. In gastric cancer cells, the inhibition of MRP4 via RNAi-mediated silencing was associated with reduced cell proliferation [44], indicating that MRP4 contributes to cancer growth, possibly via the export of PGs, which are well known to promote cancer proliferation and angiogenesis [45,46,47,48]. Furthermore, overexpression of MRP4 is associated with multidrug resistance against a number of chemotherapeutics, which are substrates of MRP4 [49]. Hence, *R*-flurbiprofen, likely acts as an inhibitor of these ABC transporters, and thereby may reduce tumor growth; however, unfortunately, in clinical studies of prostate cancer, *R*-flurbiprofen failed despite the well-documented beneficial effects of COX inhibition in cancer patients [50,51]. However, because of its low/lack of toxicity, *R*-flurbiprofen [52,53] is still an enigmatic candidate substance, for which the right indication may not be cancer but possibly immune-mediated diseases, the latter suggested by a recent study showing that *R*-flurbiprofen prevented the development of autoimmune encephalomyelitis in mice, which is a model for multiple sclerosis (MS) [30]. Although MS is likely not a PG-driven disease in humans, MRP4 may be involved in the PG-dependent transmigration of immune cells at the blood-brain barrier, where MRP4 and EP receptors are expressed [54,55]. Elucidation of its mechanism may therefore help to design related, more powerful substances. Moreover, very recently, MRP4 inhibition has been considered as a potential pharmacologic target for cardiovascular diseases [56,57]. 

In summary, our data show that *R*-flurbiprofen reduces extracellular PGs in various cancer cell lines at physiological concentrations. It inhibits the translocation of cPLA2α to its active site at the cell membrane and it blocks PG export via inhibition of the PG exporter, MRP4. These are novel mechanisms of *R*-flurbiprofen, which may unravel new indications for this enigmatic, non-toxic anti-nociceptive, anti-inflammatory, immune-modulating and anti-cancerous drug.

## 4. Materials and Methods

### 4.1. Cells and Reagents

HCA-7 (human colon carcinoma) cells were purchased from the European Collection of Cell Cultures (ECC, Salisbury, UK), HeLa (human cervix carcinoma) cells from the Deutsche Sammlung für Mikroorganismen und Zellkulturen (DSMZ, Braunschweig, Germany) and A-549 (human lung carcinoma) cells from the Cell Bank of the JCRB (Japanese Collection of Research Bioresources)/HSRRB (Human Science Research Resources Bank). HCA-7 and A-549 cells were cultured in DMEM (Dulbecco’s modified Eagle’s medium) whereas HeLa cells were cultured in RPMI medium 1640. All media contained high glucose, GlutaMAX, 10% FCS, and were purchased from Invitrogen (Karlsruhe, Germany) as well as 100 units/mL penicillin G and 100 µg/mL streptomycin. Cells were cultured at 37 °C in an atmosphere containing 5% CO_2_. *R*- and *S*-flurbiprofen were supplied by PAZ Arzneimittelentwicklungsgesellschaft (Bad Oeynhausen, Germany). The optical purity of both enantiomers was 98% (determined by stereoselective HPLC-analysis). Recombinant human IL-1 beta (IL-1β) and recombinant human tumor necrosis factor alpha (TNFα) were purchased from PeproTech (Hamburg, Germany). Calcium ionophore A23187 was purchased from Sigma-Aldrich (Seelze, Germany). cPLA_2α_ inhibitor was purchased from Calbiochem, (La Jolla, CA, USA), SC-560 and celecoxib were synthesized by WITEGA Laboratorien (Berlin-Adlershof GmbH, Germany), MK-886 was purchased from Cayman, (Ann Arbor, MI, USA) and acetylsalicylic acid was purchased from Sigma-Aldrich.

### 4.2. COX Inhibitor Screening Assay

Inhibition of COX-1 (ovine) and COX-2 (human recombinant) activity by flurbiprofen was measured using a COX Inhibitor Screening Assay Kit (Cayman Chemicals), according to the manufacturer’s protocol. SC-560, a selective COX-1 inhibitor, and celecoxib, a selective COX-2 inhibitor, were used as positive controls. The assay was started by the conversion of arachidonic acid as a substrate of the COX enzymes to PGH_2_ which is further reduced by SnCl_2_ and HCl to different prostaglandins such as PGE_2_, PGD_2_ and PGF_2α_. At the end of the reaction, the samples were centrifuged at 10,000× *g* and room temperature (RT) for 5 min, and the supernatant was diluted with methanol (1:5). In contrast to the manufacturer’s protocol, the amounts of PGs were quantified by LC-MS/MS analysis using deuterated prostaglandins as internal standards ([^2^H_4_]-PGE_2_, [^2^H_4_]-PGD_2_, [^2^H_4_]-TXB_2_, [^2^H_4_]-PGF_2α_, and [^2^H_4_]-6-keto-PGF_1α_). Then 100 µL EDTA (0.15 M), 20 µL methanol, and 20 µL internal standard solution were further added to 70 µL sample solution. LC-MS/MS sample preparation was done by liquid-liquid extraction with ethyl acetate (2 × 600 µL). After evaporation, the residue obtained was reconstituted with 50 µL acetonitrile/water/formic acid (20:80:0.0025, *v*/*v*). 

### 4.3. Analysis of Prostaglandin Levels (PGE_2_, PGD_2_ and PGF_2α_)

Cells were incubated for 24 h at 37 °C in medium containing 10% FCS. The medium was replaced with fresh medium containing either *S*-flurbiprofen (0.01–10 µM) or *R*-flurbiprofen (0.03–50 µM) and, simultaneously, IL-1β (2 ng/mL) for stimulation of A-549 cells, IL-1β (1 ng/mL) + TNFα (5 ng/mL) for stimulation of HeLa cells. HCA-7 cells produce high amounts of PGE_2_ without stimulation, therefore, we pre-incubated HCA-7 cells with medium containing either PBS or *R*-flurbiprofen (0.03–50 µM) for 1 h and replaced this medium, thereafter, with fresh medium and the same additions. This procedure ensured that all PGE_2_ produced by these cells was removed, before flurbiprofen had exerted full activity. After incubation for 16 h, the cells and the supernatant were collected and briefly centrifuged. The amount of PGE_2_ in cell lysate and the supernatant was determined using the PGE_2_ Correlate EIATM-Kit (Assay Designs Inc., Ann Arbor, MI, USA), according to the manufacturer’s protocol. In addition, the levels of PGE_2_, PGD_2_ and PGF_2α_ were measured by LC-MS/MS analysis according to previous publications [58,59].

For measurement of intracellular PGE_2_ levels cells were washed with ice-cold PBS, resuspended in Tris-CHAPS-buffer (10 mM Tris pH 7.4, 20 mM CHAPS (3-[(3-Cholamidopropyl)dimethylammonio]-1-propanesulfonate, AppliChem GmbH, Germany) , 0.5 mM EDTA (Ethylenediaminetetraacetic acid, Sigma Aldrich, Deisenhofen, Germany), 0.5 mM PMSF (Phenylmethylsulfonyl fluoride, AppliChem GmbH, Darmstadt, Germany), 1 mM DTT (Dithiothreitol, Sigma Aldrich), 2 mM Pefabloc^®^ SC, Sigma Aldrich) and sonicated with an Branson sonifier 250. Thereafter, cell-lysate was centrifuged at 14,000 rpm, 45 min, 4 °C. PGE_2_ was quantified using nano liquid chromatography tandem mass spectrometry as described before [60].

### 4.4. Human Whole-Blood Assay In Vitro

The human whole blood assay was performed as described previously [61]. Briefly, for induction of COX-1 activity, aliquots of freshly heparinized human blood (1 mL) obtained from healthy male and female informed volunteers were pre-incubated with *R*-flurbiprofen or vehicle (DMSO) for 10 min at 37 °C. Formation of thromboxane B_2_ (TXB_2_) by COX-1 was initiated by the addition of 2.5 µL calcium ionophore A23187 (20 mM in DMSO) to obtain a final concentration of 50 μM (final DMSO concentration was <1%). The reaction was terminated after 30 min by rapid cooling of the plate on ice. For induction of COX-2 activity, aliquots of freshly heparinized human blood (1 mL) were pre-incubated with 10 µL acetylsalicylic acid (ASS, 1 mg/mL in PBS; inhibits COX-1 derived PGE_2_) and *R*-flurbiprofen or DMSO for 10 min at 37 °C. PGE_2_ formation by COX-2/mPGES-1 was stimulated by adding 2 µL lipopolysaccharide (LPS, 5 mg/mL in PBS). The reaction was terminated after 24 h by rapid cooling of the plate on ice. The samples were centrifuged at 450× *g* and 4 °C for 20 min. SC-560 (COX-1 inhibitor) and celecoxib (COX-2 inhibitor) were used as controls TXB_2_ and PGE_2_ in the plasma supernatant (200 µL sample size) were analyzed as described above.

### 4.5. mPGES-1 Activity Assay

In order to investigate the impact of *R*-flurbiprofen on mPGES-1 activity in vitro, the microsomal fraction of HeLa cells was prepared. Approximately 4 × 10^6^ cells were incubated for 24 h at 37 °C in medium containing 10% FCS. The medium was removed and cells were stimulated with IL-1β (1 ng/mL) + TNFα (5 ng/mL) for 16 h. After washing with 10 mL PBS, cells were scraped in 2 mL PBS and centrifuged at 2500× *g* for 2 min at 4 °C. Cell pellets were resuspended in 600 µL potassium phosphate buffer (Kpi-buffer; 0.1 M; pH 7.4), containing 1× CompleteTM protease inhibitor cocktail (Roche Diagnostics, Mannheim, Germany), sucrose (0.25 M) and reduced glutathione (GSH; 1 mM). Samples were sonicated and centrifuged at 150,000× *g* for 1 h at 4 °C. The microsomal fraction (pellet) was resuspended in 50 µL Kpi-buffer (0.1 M, pH 7.4), containing 1× CompleteTM and reduced GSH (2.5 mM) and total protein content was measured using the Bradford method.

The mPGES-1 activity assay was performed as described by Thoren et al. [62]. The amount of PGE_2_ produced was measured by LC-MS/MS as described above.

### 4.6. Western Blot Analysis

Cells were seeded in medium containing 10% FCS and treated as indicated in the Figure legends. mPGES-1 and mPGES-2 protein was analysed in the microsomal fraction prepared as described in Section 4.5. cPGES and mPGES-2 proteins were detected in the cytosolic fraction. cPLA_2α_ and COX-1/-2 proteins were detected in whole cell lysates.

For cPLA_2α_ translocation experiments, A-549 cells were seeded at a density of 1.8 × 10^6^ cells per dish, respectively, in medium containing 10% FCS and incubated for 24 h at 37 °C. Cells were then pre-incubated with *R*-flurbiprofen (100 µm) for 30 min. Afterwards, cells were stimulated with calcium ionophore A23187 (5 µM) for 10, 30 and 60 min and simultaneously treated with *R*-flurbiprofen (100 µm). After harvesting, the cells were separated into membrane and cytosolic fractions by centrifugation. The cPLA_2α_ protein was analyzed in both fractions. MRP4 was detected in whole cell extract from HeLa, A549, and HCA-7 cells. 

Immunoblotting was performed as described previously [63]. Primary antibodies directed against mPGES-1 (rabbit polyclonal; Agrisera, Vännäs, Sweden), COX-2, cPLA_2α_ (goat polyclonal; Santa Cruz, Heidelberg), COX-1 (mouse monoclonal) mPGES-2, cPGES, MRP4 (goat polyclonal; Acris, Herford) were used. Anti-β-actin antibody (mouse monoclonal; Sigma-Aldrich) or anti-HSP90 antibody (mouse monoclonal, BD Bioscience, Heidelberg, Germany) were used as loading controls. Thereafter the membranes were incubated with secondary antibodies coupled to fluorescence dyes with an emission at 700 or 800 nm and detected with the Odyssey infrared imager.

### 4.7. cPLA_2α_ Activity Assay

cPLA_2α_ enzyme was overexpressed and isolated from Sf9-insekt cells, as described previously [64]. Multilamellar vesicles (MLVs) were prepared by drying 1-palmitoyl-2 arachidonyl-sn-glycero-3-phosphocholine (PAPC, 10 mg/mL in chloroform) and 1-palmitoyl-2oleoyl-sn-glycerol (POG, 10 mg/mL in chloroform) 2:1 under nitrogen. Vesicle buffer (134 mM NaCl, 20 mM Tris-HCl pH 7.4) was added and lipids were subjected to freezing/thawing cycles (liquid N2). For the in vitro cPLA_2α_ activity assay, small unilamellar vesicles (SUVs, 15–50 nm) were used. For this, MLVs were further downsized to SUVs by sonication for 15 min.

For the assay, 190 µL vesicle solution were mixed with DMSO (positive control), 5 µM cPLA_2α_-inhibitor (assay control) or *R*-flurbiprofen (0.1–1000 µM). One mM CaCl_2_ and 200 ng cPLA_2α_ enzyme preparation were added and the assay mixture was incubated for 60 min at 37 °C. As negative control, 190 µL vesicle solution were mixed with DMSO, 1 mM CaCl_2_ without cPLA_2α_ enzyme and also incubated for 60 min (background control for the non-enzymatic release of arachidonic acid from vesicles). The reaction was stopped by adding 1.6 mL ice-cold MeOH to each reaction tube.

For the determination of arachidonic acid by LC-MS/MS an arachidonic acid working standard within a concentration range from 0.1 to 500 ng/mL was prepared in methanol, lower limit of quantification (LLOQ) was 0.1 ng/mL. Samples for standard curve and quality control were prepared with 150 µL of blank medium, 20 µL working standards and 20 µL internal standards solution [^2^H_8_]-AA in methanol. Medium samples were prepared similarly, instead of 150 µL blank medium and 20 µL working standard 150 µL sample and 20 µL methanol were added. Arachidonic acid and internal standard were extracted with liquid-liquid-extraction. Therefore, the samples were extracted twice with 600 µL ethyl acetate. The organic phase was removed at a temperature of 45 °C under a gentle stream of nitrogen. The residues were reconstituted with 50 µL acetonitrile/water/formic acid (20:80:0.0025, *v*/*v*, pH 4.0), centrifuged for 2 min at 10,000× *g* and then transferred to glass vials (Macherey-Nagel, Düren, Germany). The content of arachidonic acid (AA) in this solution was determined by liquid chromatography-electrospray ionization-tandem mass spectrometry (LC-ESI-MS/MS). Arachidonic acid was analyzed with a Synergi Hydro-RP column (20 × 2 mm I.D, 2 µm particle size from phenomenex, Aschaffenburg, Germany) and determined with an API 4000 (Sciex, Darmstadt, Germany). Data acquisition and quantification were done using Analyst Software V 1.5 employing the internal standard method (isotope dilution mass spectrometry).

### 4.8. Silencing of MRP4

MRP4 was silenced in HeLa cells using siRNA. HeLa cells were transfected with either 10 µM MRP4 siRNA (Santa Cruz, Heidelberg, Germany) or scrambled siRNA (Ambion, Thermo Fisher Scientific GmbH, Schwerte, Germany) using siPortTM NeoFXTM Transfection Agent (Invitrogen, Thermo Fisher Scientific GmbH, Schwerte, Germany). After 24 h cells were harvested and either whole protein-extract was prepared and separated by Western blot (see above) or mRNA was extracted for quantitative RT-PCR (see below).

### 4.9. Quantitative Real-Time-PCR

Total RNA was isolated, using TRI reagent [65] and Phase Lock Gel Light tubes (5 Prime, Gaithersburg, MD, USA). RNA concentrations were determined photometrically using the NanoDrop-spectrometer (Peqlab Biotechnologie, Erlangen, Germany). cDNA was synthesized from 200 ng total RNA using the VERSOTM cDNA Kit (Thermo Fisher, ABgene, Epsom, UK). Gene specific PCR products were assayed using Maxima SYBR Green qPCR Master Mix (2×) with 10 nM ROX Solution (Thermo Fisher) on a 7500 fast quantitative PCR system (TaqMan^®^, Life Technologies, Darmstadt, Germany). Relative gene expression was determined using the comparative CT (cycle threshold) method, normalizing relative values to the expression level of RPL37A (RPL37A forward 5′-AGGAACCACAGTGCCAGATCC-3′, RPL37A reverse 5’-ATTGAAATCAGCCAGCACGC-3’) as a housekeeping gene. Primers for the determination of MRP4 (MRP4 forward 5′-GGACAAAGACAACTGGTGTGCC-3′, MRP4 reverse 5′-AATGGTTAGCACGGTGCAGTGG-3′) and RPL37A expression were synthesized by MWG (Ebersberg, Germany).

### 4.10. Statistics

The SPSS 9.01 computer software and GraphPad Prism 6.0 were used for statistical analyses. Data are presented as means ± S.E.M (standard error of the mean). IC_50_ values were analysed using a sigmoid Emax model followed by subsequent submission to univariate analysis of variance (ANOVA) and *t*-tests using a Bonferroni α-correction for multiple comparisons, α was set at 0.05. Significant *p* values are shown as: *** *p* ≤ 0.001, ** *p* ≤ 0.01, * *p* ≤ 0.05. The number of experiments is specified in the figure legends.

## Abbreviations

cPLA_2_	cytosolic phospholipase A_2_
COX-1	cyclooxygenase-1
COX-2	cyclooxygenase -2
IL-1β	interleukin-1 beta
MRP4	multidrug resistance protein 4
NSAIDs	non-steroidal anti-inflammatory drugs
PG	prostaglandin
PGES	PGE-synthases
PGT	prostaglandin transporter
15-PGDH	15-hydroxyprostaglandin dehydrogenase
TNFα	tumor necrosis factor alpha
